# Supplementary material for: Are carbon emissions trading and green financial instruments synergistic? -Comprehensive quantitative research based on content analysis
Source: PLoS One. 2024 Mar 7;19(3):e0298601. doi: 10.1371/journal.pone.0298601 (PMC10919601; doi:10.1371/journal.pone.0298601)
Supplement: S1 Data — (ZIP) [file pone.0298601.s001.zip › Data/地级市Z_F工作报告.zip!/╔╜╢1⁄2/╡┬╓▌╩╨/└┤╫╘╣1⁄2╓┌║┼í╛┬φ┐╦╩2╛▌═°í┐_2017.docx]

各位代表：

现在，我代表市人民政府向大会报告工作，请予审议，并请市政协委员和列席会议的同志提出意见。

一、过去五年回顾和2016年的工作侯卫东官场笔记

本届政府任期的五年，我们坚决贯彻党中央、国务院、省委、省政府和市委的决策部署，解放思想、抢抓机遇，从严从实、奋发有为，圆满完成了市十七届人大确定的主要目标任务。

五年来，我们凝神聚力抓发展，综合实力显著增强。经济保持较快增长，地区生产总值年均增长9.9%，人均GDP突破5万元，一般公共预算收入是2011年的1.9倍，固定资产投资年均增长17.1%，社会消费品零售总额年均增长13%，外贸保持平稳增长。人民生活水平大幅提高，城镇和农村居民人均可支配收入年均增长8.9%和9.6%，城乡收入比由1.93缩小到1.86。县域经济实现突破，德州经济技术开发区和高新技术产业开发区晋升国家级，齐河县、禹城市跻身全国百强。

五年来，我们坚持不懈调结构，发展质量不断优化。三次产业比例由11.8∶54.3∶33.9调整为10.1∶47.8∶42.1。农业现代化水平稳步提升，粮食连年丰收增产，国家现代农业示范区加快建设。工业经济发展稳健，总量持续做大，规模以上工业企业达到3022家，高新技术企业达到856家，形成4个千亿级产业集群，战略性新兴产业保持两位数增长。现代服务业异军突起，科技投入持续增加，高新技术企业发展后劲逐渐显现，银行业金融机构总资产翻一番，对经济增长的贡献率不断提高。

五年来，我们统筹协调促提升，城乡面貌深刻变化。农业转移人口市民化取得实质性进展，户籍人口城镇化率提高12个百分点。城市空间布局日益优化，陵县撤县设区，城市东进步伐加快，综合承载能力不断增强。基本公共服务向农村覆盖，美丽乡村加快建设，城乡差距进一步缩小。综合交通体系建设全面展开，德大、邯济铁路和济乐、德上高速公路建成通车，实现了县县通高速。

五年来，我们坚定不移抓改革，发展活力持续增强。认真落实市委全面深化改革部署，统筹推进823项改革任务，重点领域改革取得新突破。区域合作不断深化，全面融入国家京津冀协同发展规划，扎实推进省“一区一圈一带”战略。对外交流更加紧密，新引进世界500强企业7家，国际友城和友好合作城市达到11个，与欧美、日韩、东南亚等国家的交流合作日益深化。

五年来，我们倾心尽力保民生，社会事业全面进步。民生领域累计支出1168.12亿元，占财政支出的80.32%。集中破解了一批群众关心的民生问题。社会保障体系日趋完善，基本养老保险、医疗保险、大病保险和社会救助基本实现全覆盖。城乡一体的治安防控体系不断完善，平安德州建设成效明显。教育、医疗、文化等社会事业加快发展，市博物馆、档案馆、大剧院投入使用，南运河德州段列入世界文化遗产名录。

2016年是本届政府的收官之年，我们坚持“创新、协调、绿色、开放、共享”发展新理念，以推进供给侧结构性改革为主线，攻坚克难，主动作为，经济发展稳中向好，实现了“十三五”良好开局。

（一）经济保持平稳增长，综合实力进一步增强。经济运行处在合理区间，实现地区生产总值2933亿元，增长7.2%；一般公共预算收入183.5亿元，同口径增长7.1%；固定资产投资2537.8亿元，增长13.4%，增幅居全省第3位；社会消费品零售总额1395亿元，增长10.9%；外贸进出口211.23亿元，增长7.3%。城镇新增就业6.5万人，年末城镇登记失业率为2.64%；居民消费价格涨幅1.8%，处于温和上涨区间。城乡居民人均可支配收入分别达到22760元和12248元，增长8.2%和8.7%。

（二）推进供给侧结构性改革，发展动能加快转换。认真落实“三去一降一补”，新的动能在成长，向好因素在累积。产能化解进展良好，平板玻璃、水泥、炼油等过剩产能化解率79.16%，关停化工企业139家，淘汰落后生产线12台（套），超额完成省下达的化解过剩产能任务。严厉打击制售“地条钢”等违法行为。商品房去化周期由年初的21.6个月降至12.9个月，库存水平趋于合理。企业杠杆率不断下降，全市规模以上工业企业资产负债率35.3%，低于全省平均水平。加快推动企业股权多元化，完成规范化公司制改制企业308家，新增新三板挂牌企业6家，直接融资303.79亿元。企业成本稳步降低，从税费、融资、电价、社保等方面综合施策，为企业减负4亿多元，“营改增”惠及2.3万户。着力补齐发展短板，出台一系列支持发展的意见，扎实开展“进企业、解难题、促发展”工作，解决企业困难456个。

新动能加快形成。新认定省级以上高新技术企业60家、企业研发平台120个；高新技术产业投资576.14亿元，增长31.7%；高新技术产业产值3132.87亿元，增长10.43%，增幅居全省第3位，占规模以上工业产值29.44%，比年初增长1.39个百分点。全面实施人才引进“黄金30条”，引进省级以上高层次人才241人，1000家企业与587个高校院所建立了合作关系，建成人才公寓56.49万平方米，德州创新谷、应用技术研究院开工建设。产业升级步伐加快，工业技改投资792亿元，占工业投资的55%。电子商务、健康养老、文化旅游、科技金融等现代服务业加快发展，服务业占比提高1.8个百分点，成功列入国家产融合作试点城市和全国物流标准化试点城市。

（三）深化对外合作，协同发展成效显著。抢抓国家京津冀协同发展、“一带一路”和省“一区一圈一带”战略机遇，向北对接京津，向南融入省会，向东连接出海口。积极争取省委、省政府出台专项政策，支持我市融入京津冀协同发展。生态科技城被国家确定为北京非首都功能疏解重点承接平台。坚持招商引资与招才引智并重、产业项目与社会项目并举，签约京津冀亿元以上产业项目197个，新开工109个，北汽宝雅、联孚客车、华日家具、富士康、国电投、汇源等一批重点项目加快建设。京沪高铁“德州号”正式开通，成功举办首届京津冀鲁技术交易大会、资本交易大会、山东第九届“海洽会”暨首届“智汇德州”人才创新创业周三大活动，区域产业融合发展势头强劲。积极融入省会城市群经济圈，基础设施日益完善，济齐黄河大桥顺利合龙。与山东大学的合作全面深化，生物工程、晶体材料、智能装备研究院等11个合作项目落户我市。浪潮大数据中心、齐鲁制药产业园、汉格威新能源汽车电控等58个项目顺利推进。平原县与青岛港的海铁联运专线开通，海尔物流园、青啤新增产能等项目进展较快。加强与东盟国家的产业合作，成功举办中国—东盟中小企业合作会议，东盟国际城、马来西亚齐力集团等东盟项目全力推进，恒源石化、奥冠电池等企业成功拓展东盟业务，东盟已成为我市第一大贸易伙伴，进出口增长47.2%，德州正努力打造中国北方对接东盟的重要节点城市。

（四）全面深化改革，发展活力不断释放。深入实施52项国家、省级改革试点，研究审议改革方案30个。深化“放管服”，对11项市级权力实行“市县同权”，在禹城市和齐河县试点31项事权审查和批准相分离。全面实施“五证合一、一照一码”，市场主体达到32.57万户，增长13.4%。市级政务服务平台上线运行，实现省市县互联互通。整合建立统一的公共资源交易平台。扎实推进财税金融改革，用好市级股权投资引导基金，发起设立城市建设、现代农业、生物产业等专项基金，引入社会资本。投融资体制改革成效明显，新组建了4家市级国有资本运营公司，指导各县（市、区）完善投融资模式，通过政府购买服务、PPP、融资租赁等方式，提升了融资发展的能力。新型农村合作金融改革有序实施。加快农村改革步伐，农业适度规模经营稳步推进，累计流转土地面积310万亩，培育家庭农场2069家。供销社综合改革和农村产权制度改革取得阶段性成效。机关事业单位改革有序推进，教育、医疗综合改革不断深化。不动产统一登记制度基本建立。党政机关公车改革如期完成，压减公务用车3391辆，公车购置及运行维护费用减少21%。司法体制改革和社会治理体制改革继续深化。

（五）打造“放心农场”，现代农业转型提速。编制完成《德州市现代农业发展总体规划》，加快实施现代农业十大工程。粮食继续保持高产，达到179亿斤，调减籽粒玉米20.6万亩，新增设施蔬菜10.2万亩。“三品一标”认证基地面积达到654万亩，农产品质量安全抽检合格率98%。成功举办首届德州名优特新农产品对接京津冀博览会，我市农产品优质、安全品牌形象得到提升。新增国家级农业龙头企业1家，新建扩建华大锐护、新希望、中节能农光互补、昌盛日电、广州温氏等一批高端农业项目。深入推动农业国际合作，与德国巴斯夫集团、荷兰瓦赫宁根大学合作共建项目有序推进，世界500强美国雅培·恒天然醇源牧场一期投产运营。

（六）推动协调发展，新型城镇化步伐加快。扎实推进“三个市民化”，完成9万农业转移人口和5.6万城中村、城边村居民市民化任务，常住人口和户籍人口城镇化率分别达到53.77%、42%。深入实施新型城镇化试点，武城县产城融合就地城镇化试点和齐河县户籍制度改革得到省委、省政府充分肯定，禹城市土地征收制度改革顺利推进。积极推动城市有机更新，中心城区改造“三无小区”79个，12973户群众改善了居住条件；全市新开工棚户区改造18981套，开工率115%。“两区同建”扎实推进，新建续建农村新型社区150个，80%以上实现集中供暖。完成农村危房改造4611户，改厕14.6万户。重大基础设施建设进展顺利，石济客专等项目加快推进，南水北调续建配套工程在全省考核第一名。

（七）补齐发展短板，民生事业持续改善。脱贫攻坚精准推进，投入资金4.47亿元，实施产业扶贫项目1342个，减少贫困人口5.6万，超额完成年度脱贫任务。社会保障制度不断健全，保障覆盖范围持续扩大，城乡低保标准进一步提高。教育事业实行饱和式投入，完成投资59亿元，是过去6年的总和，“全面改薄”开工校舍面积246.35万平方米，解决“大班额”开工校舍面积245万平方米，竣工率分别居全省第1位、第4位，178所新建学校竣工，增加学位10万个，新招聘教师3193名，农村学校和师资力量得到加强。公共卫生事业加快发展，市人民医院新门诊楼、市中医院病房楼、市妇女儿童医院病房楼加快建设，市残疾儿童康复中心完成主体工程。政府出资为70名听障儿童植入人工耳蜗。全面二孩政策平稳实施。文化事业加快发展，134个乡镇综合文化站全部达到国家三级以上标准，建成村级综合文化服务中心1992个。养老服务加大供给，新建城市社区日间照料中心26处、农村幸福院120处，新增养老床位4100张。农村公路安全防护工程顺利完成。平安德州、食安德州建设不断加强，社会治安持续向好，安全生产总体平稳。

（八）科学精准施策，生态环境显著改善。牢固树立绿色发展理念，全力打好生态环境治理攻坚战。坚持依法治理，制定首部德州市政府规章《大气污染防治管理规定》，在全省率先出台环保评估负面清单。采取“压煤、抑尘、控车、除味、增绿”五大措施，市县两级财政安排2.65亿元，全域化大力度推进散煤清洁化治理，初步建立起清洁煤配送体系，走在全省前列。完成2759台10吨以下锅炉淘汰和清洁能源替代，44台大型锅炉实现超低排放改造。华能德州电厂、华鲁恒升、黑马集团、莱钢永锋等全市大批企业在治污上舍得投入，为改善空气质量作出了重要贡献。狠抓城市扬尘、机动车排气综合治理，效果显著。全年蓝繁天数达到182天、增加29.1%，SO2、NO2、PM10、PM2.5分别改善17.1%、13%、13%、19.8%，获得省生态补偿资金2710万元。我市年度空气质量改善率、PM2.5改善幅度、获得生态补偿资金实现了三个“全省第一”。深化流域污染治理，主要河流水质稳定达到V类标准。加快生态修复，建设人工湿地28处，植树1912万株，启动实施运河开发区生态修复三年规划。

（九）坚持从严从实，政府自身建设不断加强。自觉接受人大及其常委会的监督，加强与政协的民主协商，对提出的意见建议件件落实，对形成的调研成果充分采纳。共办理人大代表建议341件、政协委员提案507件。虚心听取各民主党派、工商联、无党派人士和社会各界意见。坚持依法行政，以政府规章形式出台《德州市重大行政决策程序规定》，全面规范政府决策行为。实施电子政务提升工程，大力推进政务公开。加大审计监督力度，强化对权力的制约。全面落实从严治党主体责任，严守党的政治纪律和政治规矩，从严从实抓好政府系统干部队伍作风建设，扎实开展“两学一做”学习教育，严格落实中央八项规定精神，坚决纠正“四风”。全市纪检监察机关立查案件2082件，党纪政纪处分1934人，党风廉政建设和反腐败工作取得积极成效。

各位代表，五年来的发展和进步，是我们全面坚持党的领导，认真贯彻落实党的路线方针政策的结果，是省委、省政府大力关心支持的结果，是市委坚强领导的结果，是市人大、市政协监督支持的结果，是全市上下共同努力的结果！在此，我代表市政府，向全市人民，向各位人大代表、政协委员、离退休老同志，向各民主党派、工商联、无党派人士和各群众团体，向驻德部队官兵，向中央、省驻德单位、各级新闻媒体，向所有关心支持德州发展的海内外朋友，致以崇高的敬意和衷心的感谢！

我们也清醒地认识到，当前我市经济社会发展中还存在一些突出问题：经济下行压力依然较大，财政增速放缓，部分行业、企业经营困难，投资乏力还没有根本改善；企业整体创新能力和竞争力还不强，高端产品比重仍然较低；能源结构仍不够合理，资源环境约束趋紧；民生短板仍比较突出，部分群众生活还很困难，基本公共服务与群众期望还有较大差距；发展环境还需进一步优化，部分政府工作人员推动新发展能力不足，庸懒散、不作为、乱作为的问题仍然存在，高效服务的意识有待提高；隐形变异的“四风”问题仍然不同程度存在。对上述问题，我们一定高度重视，加大工作力度，积极予以解决！

二、今后五年的任务

各位代表，德州已经站在新的历史起点上，刚刚闭幕的市第十五次党代会描绘了德州未来发展的宏伟蓝图，指明了前进方向。我们要按照市委的决策部署，践行新理念，实现新发展，努力开创协同发展新局面！

未来五年，我们肩负重大责任。全面建成小康社会的奋斗目标要在未来五年实现，经济社会发展水平要达到新高度。“十三五”规划要全面落实，产业结构调整要取得明显成效。融入京津冀等区域发展战略要有阶段性突破。脱贫攻坚、生态建设等一系列重大任务要全面完成，协同发展示范区建设要在未来五年取得重要成果。

未来五年，机遇与挑战并存。我国经济增速进入换挡期，结构调整进入阵痛期，前期刺激政策进入消化期，不确定、不稳定因素增多。传统的增长模式难以为继，生态环境的承载能力日趋严峻，城市间对新兴产业和高端人才的争夺日趋激烈。同时，更应该看到，宏观政策在日益优化，区域发展战略的红利在持续释放，新产业、新模式在不断涌现，“大众创业、万众创新”在持续激发活力，我们必须尽快适应新常态，落实新理念，学习新本领，实现新突破。

未来五年，我们充满信心。德州区位优势得天独厚，战略机遇十分难得，经过多年奋斗，我们已经积累了近3000亿的地区生产总值，3000多家规模以上工业企业，以及较强的科技创新实力和人才储备，发展潜力巨大，机遇大于挑战。天道酬勤！只要我们坚持以习近平同志为核心的党中央坚强领导，认真贯彻落实上级的各项决策部署，紧扣德州发展实际，机遇不丧失，精神不懈怠，实干会干加苦干，就一定能够谱写德州协同发展的美好未来！

今后五年政府工作的指导思想是：全面贯彻党的十八大、十八届历次全会和即将召开的党的十九大精神，深入学习贯彻习近平总书记系列重要讲话精神，统筹推进“五位一体”总体布局，协调推进“四个全面”战略布局，以新发展理念为引领，以供给侧结构性改革为主线，深入实施协同发展战略，加快建设协同发展示范区，努力提高人民生活水平，促进经济平稳健康发展和社会和谐稳定，圆满完成“十三五”规划目标，奋力夺取全面建成小康社会新胜利，以优异成绩迎接中国共产党成立100周年。

今后五年的主要任务是：

——全面转变经济发展方式。紧紧扭住发展这个第一要务不动摇，地区生产总值要突破4000亿元，比2010年翻一番，一般公共预算收入达到260亿元，投资保持两位数增长。更加注重产业结构调整，未来五年要把经济发展的动力进一步调整到依靠技术进步和创新驱动上来，大力培育高新技术企业，大力引进战略性新兴产业，大力支持企业转型升级，大力淘汰落后产能。更加注重发展的平衡性、协调性和可持续性，工业化、信息化、城镇化、农业现代化要同步发展、融合发展，构建起现代产业发展新体系。不片面追求发展速度的增长，更着重于发展质量、发展效益和城市综合竞争力的提升。加快壮大县域经济，缩小县域发展差距，实现更加均衡，一般公共预算收入过20亿的县（市、区）要达到6个。更加注重培育开放型经济发展新优势，推进全方位对外开放，建立“对内对外双向开放”新格局，更好地融入区域经济一体化，广泛开拓国内国际市场，培育一批具有国际合作能力的优秀企业和企业家。

——全面提高人民生活水平。牢固树立以人民为中心的发展思想，使“学有优教、住有宜居、老有颐养、病有良医、困有相助”，让德州人民共享改革发展的成果。教育资源要进一步优化，基础教育要更加均衡，高中教育要进一步扩容，职业教育要大力发展，特别是农村教育要大力提高保障能力。要保持房地产业稳定，基本住房保障要增加投入，五年内城镇棚户区、城中村、城边村改造基本完成，三年内中心城区“三无小区”改造全部完成。美丽乡村建设要进一步提速，全市农村危房改造基本完成。大力发展养老事业，鼓励各类养老服务机构发展，福利保障型养老要增加公共投入，改善型养老要给予政策支持。医疗卫生水平尽快提升，加强与京津地区高水平医院的合作，在增加硬件投入的同时，下大力气提高医务人员的治疗水平，将医师培训逐步纳入财政保障。要大力发展中医，使优秀的中国传统医学更多地惠及群众。要从严从实全面完成脱贫攻坚任务，建立长效机制。不断提高社会保障标准，实施全民参保登记计划，充分保障就业创业，更加关注和解决好困难群体的生活问题，让社会充满关爱和温暖。

——全面推动城乡统筹发展。高度重视规划的引领作用，按照“全域统筹、产城融合、协同发展 ”的思路，开展新一轮城市总体规划修编，优化城市空间布局。中心城区要加快提升，行政区划要进一步调整，充分释放和优化城市资源。东部城区要加快崛起，老城区要提升功能，运河开发区要全力修复生态，陵城区要全面融入。各县城和中心镇要加快建设，提升城市面貌，完善城市功能，成为吸纳农村人口转移的主阵地。要大力发展特色小镇建设，积极对接政策资源和市场资源，立足乡村特色和现代生活需求，因地制宜高水平建设特色小镇群，提升乡村旅游档次，吸纳农村人口就业，努力打造周边城市假日休闲目的地。加快新型城镇化步伐，全面放开户口迁移政策，探索更加有效的农业转移人口市民化路径。要坚定不移推动农业供给侧结构性改革，大力建设“放心农场”，保持粮食产量稳定，保障农产品质量安全，稳妥优化粮食和经济作物比例，积极引进农业领军企业和新的生产方式，培育新型职业农民，在全国率先建成生态信任农业先行示范区，推动德州农业发生深刻变革。

——全面加强社会建设。大力弘扬公平正义，积极践行社会主义核心价值观，繁荣发展文化事业，加强社会信用体系建设，使“我文明德州文明”蔚然成风。提高公共决策能力，建立开放的公共决策体系，使公共权力广泛接受社会监督。更加重视城市公共安全，对各类风险源实施全覆盖管理，运用大数据等技术，建立起网格化的城市公共安全体系，保障人民群众生产生活安全。以最大的决心、最大的力度持续推进生态环境建设，努力建设京津南部、山东北部重要的生态功能区。不断改善能源结构，降低工业排放，大规模植树造林，持续优化空气质量。环境治理和生态建设既是攻坚战，也是持久战，不可能一蹴而就，但只要我们坚守绿色发展理念不动摇，以钉钉子的精神，一件事接着一件事办，一年接着一年干，总有迎来绿水青山的那一天！

三、2017年的重点工作

今年是全面落实市第十五次党代会精神的开局之年。做好今年工作，必须坚持稳中求进工作总基调，保持战略定力，稳定社会预期，切实防范风险，努力在稳的前提下奋发有为，全面展开各项工作。

今年我市经济社会发展的主要预期目标为：地区生产总值增长7.5%左右，固定资产投资增长12%左右，社会消费品零售总额增长11%左右，进出口稳定增长。城镇登记失业率控制在3.5%以内。居民消费价格上涨3%左右。一般公共预算收入增长7%左右。城镇和农村居民人均可支配收入分别增长8%左右和8.5%左右。

主要做好以下重点工作：

（一）全力推进协同发展示范区建设

推动区域经济一体化。融入京津冀协同发展和省“一区一圈一带”战略要进一步提速升级，向纵深推进。加快引进建设高端产业项目，确保每个县（市、区）年内新开工投资过亿元的产业项目不少于5个。支持北汽宝雅、晶华集团等企业与央企进行产业合作。要更加注重新兴产业和实体项目的引入，建立精准招商体系，着力突破战略性新兴产业，尽快形成产能和效益。要严把项目准入关，不符合环保标准的坚决不上，有重大安全风险的坚决不上，高能耗、高污染、低水平的坚决不上。要更加注重社会项目的合作，全力推动高端教育、医疗和文化项目的引入，在政策上给予充分保障。要更加注重人才和技术创新的作用，继续办好京津冀鲁资本交易大会、技术交易大会和“智汇德州”创新创业周，发挥好这些平台的作用，千方百计吸引资本、技术和人才向德州汇聚。要继续加强与大院大所的合作，力争再引入3—5家重点高校产业研究院。优化人才环境，坚持引进外来人才与培养本地人才并重，引进产业型、科技型人才与引进教育医疗等公共服务类人才并重，引进高层次人才与引进技能型、实用型人才并重，引进人才与长期扶持人才并重，努力增加城市人才总量。全年新建设人才公寓60万平方米，鼓励兴办“乡村人才公寓”，新设立博士后科研工作站5个以上，科技孵化器和众创空间面积达到200万平方米。

构架城市开放新格局。以开放引领德州实现新发展，打破保守思想和小成即满的观念，以更宽阔的视野和更高的标准抓好发展，全面提升经济工作的本领。主动对接“一带一路”战略，强化与东盟国家的产业合作，以中小企业为重点，在新型食品、新能源、装备制造等领域，坚定不移走出去，今年市政府将组织100家企业出访东盟国家。要深化我市与欧洲国家在现代农业领域的合作，积极引入先进技术，在德城区、经济技术开发区、陵城区、临邑县、齐河县等率先建设30个百亩级“智慧农业大棚”，打造高端智慧农业走廊。支持我市企业拓展非洲和中东市场，大力建设海外仓，开展跨国经营和境外投资，在太阳能利用等领域实现新突破。精心筹办国际太阳能十项全能竞赛，加快太阳能“德州小镇”建设，进一步拓展在新能源领域的国际合作。要加强与各友好城市的关系，全方位开展文化、教育等人文领域的交流。

（二）突出发展实体经济，支持企业做大做强

营造良好发展环境。实体企业是经济的命脉，企业家是最宝贵的资源。要深化“政府创造环境，企业创造财富”的理念，建立“亲”“清”新型政商关系，用心帮助企业降低成本、开拓市场、招募人才。市县两级都要积极行动起来，常态化推进“进企业、解难题、促发展”工作，真心实意帮助企业，千方百计扶持企业。要鼓励企业家干事创业，做大主业，筑牢实体经济发展的根基，在全社会营造尊重企业家的浓厚氛围。全市企业在安全生产、节能减排等方面也要坚决落实主体责任，下大气力抓好整改提升，共同促进安全发展的良好局面。

全面实施“三航计划”。经济新常态下，既要发挥市场在资源配置中的决定性作用，也要更好地发挥政府作用，勇于担当。当前，融资难已成为实体经济普遍存在的问题，许多企业深陷担保圈担保链而举步维艰。今年政府要下更大的气力帮助企业破解融资难题，引导金融资源精准投向实体经济和小微企业。发挥政府“黄金信用”的作用，主动出击，综合运用政策性金融工具，在全市选择30家大型骨干企业，实施“护航计划”，选择50家高成长性企业，实施“远航计划”，选择100家科技型中小微企业，实施“启航计划”，针对不同阶段的企业，由国有资本运营公司、担保公司等综合开展资金过桥、贷款担保、股权投资、风险补偿等服务，积极助推我市优质企业发展。有序开展“破圈断链”，努力化解不良贷款，守住不发生系统性区域性金融风险的底线。

全面深化科技创新工作。组建市科技创新委员会，统筹推进创新驱动各方面工作，全面推进“大众创业、万众创新”。要落实已出台的各项扶持政策，加快科技服务业的发展，财政支持为全市所有规模以上工业企业培养技术经纪人，提高企业参与技术转移的能力。健全科技金融体系，支持市科技成果转化基金与各类天使投资、风险投资等开展合作，助力中小微企业成长。加快山东省技术转移转化中心建设，支持德州高新区建设中央创新区。再组建7个市级产业技术创新联盟，新认定高新技术企业100家左右，新增企业科技创新平台120个。

大力推动企业转型升级。全面推进150个工业转型升级导向计划项目，引导企业完善现代企业制度，完成规范化公司制改制330家以上。支持企业对接资本市场，力争3家企业主板上市、6家企业新三板挂牌，全年实现直接融资300亿元以上。支持企业通过并购重组、参股控股、技术合作等各种方式开展对外合作。积极推广智能化生产、个性化定制、装配式建筑等新业态。着眼“提升一批、规范一批、淘汰一批”，全面展开低速电动车整治工作。严格执行安全生产、环保、能耗、质量等标准，倒逼重点行业过剩产能退出，稳妥施策，加快“僵尸企业”市场出清。

深入实施质量强市和品牌战略。逐行业开展质量提升行动，大力倡树“工匠精神”，用一流质量创造竞争优势。引导企业深化品牌意识，加大品牌投入，加快培育一批自主品牌、特色品牌和国际化品牌。巩固升级“老字号”，努力使德州扒鸡、乐陵小枣、德州黑陶等老品牌擦得更亮，培育壮大“新字号”，使泰山、皇明、景津、希森、保龄宝、丽驰等新品牌群叫得更响、走得更远，使“德州制造”成为品质的保障，成为城市靓丽的名片。

（三）深入推进农业供给侧结构性改革

德州农业要实现由大到强，必须把农业供给侧结构性改革作为主线，以建设周边城市“放心农场”为目标，大力发展现代农业，这是德州农业发展壮大的根本出路！

优化农业结构。继续开展绿色增产和粮食高产创建，确保粮食生产稳定。适应市场需求，扩大优质专用主粮面积，实现优质优价。着力推进种植业“一减两扩”，调减籽粒玉米种植面积，扩大优质蔬菜和特色林果种植规模，使粮经比例达到合理范围。推进畜牧业“一稳两增”，稳定生猪养殖规模，适度增加草食牲畜和家禽养殖数量。推进生产经营“一降两升”，以规模化经营降低农业生产成本，推进物质装备条件升级和资源利用方式升级。

转变农业发展方式。积极引进市场要素进入农业领域，推动一产接二连三。规划建设20个市级“放心农场”、10个规模化高效农牧业示范基地和20个精品特色农业示范村镇。大力引进现代农业项目，发展精准农业、精深加工，各县（市、区）每年至少新增2家农业产业化龙头企业，以先进的技术和成熟的运营模式带动农业转型。加快构建中央厨房产业体系，建立以服务周边大城市为导向的现代农业产业链。健全农产品质量安全可追溯体系，打造一批地理标志农产品和区域特色品牌，推进整建制创建省级农产品质量安全市。大力发展乡村旅游、农村电商等新业态。推行绿色生产方式，大力发展节水农业，实施化肥、农药零增长行动，加强耕地保护与地力提升，构建农业可持续发展新格局。

激发农业农村内生动力。出台现代农业发展政策体系，鼓励农业经营主体转型发展，大力培育新型职业农民。深化农村产权制度改革，激活各类资源要素，增加农民产权收益。积极推进适度规模经营，大力发展各类新型经营主体，组织评选100家市级示范社。稳妥推进农村金融创新，深化供销社综合改革，做大做强为农服务主平台。

（四）全面推进城乡建设

推进新型城镇化。深化户籍制度改革，完成12.1万人市民化目标。全面推广武城县城镇化试点经验，探索“两区同建”农村居民就地市民化路径。以全域眼光完善城镇规划体系，推进交通设施、产业布局一体化，积极培育中小城市和特色小镇。实施棚户区改造46495户，提高货币化安置比例。改造农村危房3000户，新建改造农村公路1200公里，进一步缩小城乡差距。

提升城市建设管理水平。投资261亿元实施6大类、81个中心城区城建项目，重点推进国际会展中心、市民中心、金融港等十大城市功能支撑工程。实施三八路下穿京沪铁路隧道拓宽、市人民医院周边地下停车场等重点项目，集中破解停车难、通行难等长期困扰群众生活的突出问题。启动城区环境“三年提升行动”，再投资3.24亿元，改造中心城区75个“三无小区”；加强公共艺术空间设计，提升城市文化品位。深入开展城市违法建设治理行动，严厉打击私搭乱建行为，提高城市精细化、智能化管理水平。完善城市现代交通体系，启动中心城区东西快速交通建设和轨道交通论证，大幅度增加投入，中心城区再增加300辆新能源公交车，建设充电设施，提高市民公交出行率。

（五）着力抓好生态环境建设

牢固树立“绿水青山就是金山银山”的理念，一定要让群众喝上干净的水，呼吸到新鲜的空气，看得见蓝天白云。

坚持不懈抓好污染治理。实行“逐月调度，逐县落实，网格化推进”，锲而不舍增加“蓝天白云、繁星闪烁”天数，全年力争达到195天以上，PM2.5再改善15%，PM10再改善18%。全面推行河长制，确保主要河流水质稳定达标，整治河道，优化管网，全面消除城区黑臭水体，加强重要饮用水源及南水北调水质安全保障。继续在全域推广清洁煤125万吨，加大对困难群众的补贴力度，在农村大力推广新能源，继续扩大城区集中供热面积。加强扬尘整治和渣土运输管理，规模以上工地全部安装在线监测和视频监控。加强挥发性有机物治理，加快油品质量升级，强化高排放车辆监管。依法治理“散乱污”企业，全面清理整治砖瓦窑等窑炉。推进排污许可管理，实施工业源全面达标排放。力争全社会煤炭消耗再压减40万吨，全面淘汰10吨以下燃煤锅炉。

坚持不懈抓好生态修复。重点实施运河开发区生态修复三年规划，集中实施64个修复项目，棚户区改造加快实施，小散堆场全面整治，着力解决重型车过境穿城等问题。启动建设德州大运河生态林场和西陈沟湿地公园，3年内在运河开发区集中连片造林7500亩，今年启动种植3100亩，建设城市森林，与重工业区形成有效的生态间隔。实施“再建千亩湿地”计划，在全市展开重污染土壤治理和修复试点，高标准新建农田林网35万亩、储备林基地10万亩，植树2500万株。

（六）加强社会事业建设

坚决打赢脱贫攻坚战。在全面建成小康社会的道路上，不能让一个人掉队。今年我市要实现3.98万省定标准贫困人口脱贫，基本完成脱贫任务。深入实施贫困村“一村一品”产业推进行动，提高产业扶贫项目质量和效益。突出贫困村基础设施、公共服务和集体经济提升，完成276个省扶贫工作重点村“五通十有”工程。农村低保标准全部达到省定扶贫标准线。落实就业、保险、低保、救助等措施，统筹解决城市贫困问题。

推动优质教育资源共享。支持德州学院建设高水平综合性大学，支持职业教育蓬勃发展。全面解决城镇中小学“大班额”问题，按计划完成农村薄弱学校改造任务。新建、改扩建学校115所，开工校舍面积45.78万平方米，新增学位9.9万个，新建幼儿园72所、改扩建76所，实现县域义务教育均衡发展全覆盖。开工建设市属高中、高级师范学校、一中扩建、实验中学规范化工程。积极推进校长职级制改革和教师“县管校聘”改革。

大力发展卫生健康事业。持续加大投入，统筹布局卫生资源。加快建设分级诊疗服务体系，健全家庭医生签约服务机制。推进公立医院建立法人治理结构。加强重点学科建设，实施“青年医学专家培养计划”，选拔培养优秀后备人才。发展医养融合型养老机构，开工建设市级养老示范项目，新建城市社区日间照料中心10处、农村互助幸福院80处，新增养老床位3000张。认真落实全民健身国家战略，提升竞技体育水平，培养健康生活方式。

健全完善社会保障体系。新增城镇就业6.1万人。全面实施机关事业单位养老保险制度。建立职工大病保险制度，完善居民大病保险政策，实现居民医疗保险市级统筹。统筹城乡特困人员救助供养。发展老龄事业，保障妇女权益，关心青少年成长，加强未成年人保护和困境家庭保障，关爱留守儿童，做好残疾人基本公共服务和残疾预防，支持慈善事业发展。无论财政压力有多大，民生支出只增不降，惠民实事只加不减。

扎实推进文化建设。建设高水平市级文化中心，提升图书馆、文化馆等各类市级文化设施水平，加快县级国家综合档案馆和档案信息化建设，开工建设山东自然博物馆。加强苏禄王墓、东方朔墓等文化遗产保护，推进非物质文化遗产传承进校园。加快文化产业转型升级，积极寻求对外合作，引进微电影小镇等有竞争力的文化产业项目。支持媒体深度融合发展，支持党报党刊做大做强，推动市县广播电视台改革。完善现代公共文化服务体系，努力探索有德州特色的文化产业发展之路。

加强和创新社会治理。深化平安德州建设，完善立体化、信息化社会治安防控体系，依法惩治违法犯罪行为。落实安全生产责任，强化过程监管，坚决防止重特大事故发生。严防严管严控食品药品安全风险，加强市场管理，保证群众吃得放心、安心。做好信访工作，及时回应群众合理诉求，妥善化解各类矛盾，维护社会稳定。继续做好双拥共建工作，支持国防和军队改革，加强国防后备力量建设，推进军民深度融合发展。

四、落实全面从严治党各项要求，加强政府自身建设

严格依法履职。全面提高政府工作人员法治思维和依法行政能力，依法接受人大及其常委会的法律监督和工作监督，主动接受人民政协的民主监督，密切与各民主党派、工商联、无党派人士和人民团体的联系沟通，自觉接受司法监督、舆论监督和社会监督。完善政府法律顾问制度，做好行政复议和行政应诉工作，确保权力在阳光下运行。市政府是德州人民的政府，无论做什么决定、办什么事情，都要于法有据，都要充分体现群众意愿，都要接受群众监督。

切实转变职能。继续深化简政放权、放管结合、优化服务，整合审批流程，加强事中事后监管，做到该放的放开、该管的管好。努力提高行政效率，密切联系群众，转变工作作风，善于运用先进技术推动政务公开。推行行政决策科学化、民主化、法治化，落实重大行政决策程序，完善公共参与机制，提高专家论证和风险评估质量，加强政府重大行政决策合法性审查，严格行政决策责任追究。坚决打破隐形权力的玻璃门，坚决惩治伤害群众的行政行为。

落实主体责任。牢固树立“四个意识”特别是核心意识和看齐意识，把政治纪律和政治规矩挺在前面，严肃党内政治生活，从严从实抓好市政府党组和政府系统党的建设，切实担负起全面从严治党的主体责任。严格落实《准则》《条例》和中央八项规定，驰而不息纠正“四风”，认真落实上级各项决策部署，确保令行禁止、政令畅通。坚决制止一切形象工程、政绩工程，反对任何统计数据造假，严肃查处不作为、慢作为行为。深化述职述廉，推进审计监督全覆盖，强化行政监察和行政执法监督。坚持开正门、堵旁门、关后门，消除权力运行灰色地带，破除各种形式“潜规则”。严格公务员队伍管理，着力解决群众身边的不正之风和腐败问题。完善正向激励和容错纠错机制，强化各级国家公务人员的责任担当，保持奋发有为的精神状态，营造风清气正的政治生态。

各位代表！一代人要有一代人的作为，一代人要有一代人的贡献！新的征程已经开启，德州大地春潮涌动，德州人民满怀期待，让我们更加紧密地团结在以习近平同志为核心的党中央周围，高举中国特色社会主义伟大旗帜，在中共德州市委的坚强领导下，弘扬新时期长征精神，撸起袖子加油干，共同创造德州美好的未来！奋力夺取全面建成小康社会新胜利，以优异的成绩迎接党的十九大和省第十一次党代会胜利召开！
